# In Vitro Comparison of Antibacterial and Antibiofilm Activities of Selected Fluoroquinolones against *Pseudomonas aeruginosa* and Methicillin-Resistant *Staphylococcus aureus*

**DOI:** 10.3390/pathogens8010012

**Published:** 2019-01-24

**Authors:** Majed M. Masadeh, Karem H. Alzoubi, Wesam S. Ahmed, Aisha S. Magaji

**Affiliations:** 1 Department of Pharmaceutical Technology, Jordan University of Science and Technology, Irbid 22110, Jordan; 2 Department of Clinical Pharmacy, Jordan University of Science and Technology, Irbid 22110, Jordan; khalzoubi@just.edu.jo (K.H.A.); wisamyejo@yahoo.com (W.S.A.); 3 Department of Applied Biology, Jordan University of Science and Technology, Irbid 22110, Jordan; magajiaisha22@gmail.com

**Keywords:** biofilm, fluoroquinolones, methicillin-resistant *Staphylococcus aureus*, planktonic, *Pseudomonas aeruginosa*

## Abstract

An in vitro overview of the inhibitory effects of selected fluoroquinolones against planktonic and biofilm cells of the methicillin-resistant *Staphylococcus aureus* (MRSA) strain American type culture collection (ATCC) 43300 and the *Pseudomonas aeruginosa* strain ATCC 27853 was carried out. Biofilm cells of both strains were less susceptible to the selected antibiotics than their planktonic counterparts. In addition, certain antibiotics were more effective against biofilm cells, while others performed better on the planktonic cells. Against *P. aeruginosa*, ciprofloxacin was the most potent on both planktonic and biofilm cells, whereas ofloxacin was the least potent on both biofilm and planktonic cells. Moxifloxacin and gatifloxacin were the most potent against both planktonic and biofilm MRSA bacteria, however, not in the same order of activity. Norfloxacin was the least active when tested against both planktonic and biofilm cells. The results of this work are expected to provide insight into the efficacy of various fluoroquinolones against MRSA and *Pseudomonas aeruginosa* biofilms. This study could form the basis for future clinical studies that could recommend special guidelines for the management of infections that are likely to involve bacteria in their biofilm state.

## 1. Introduction

A biofilm represents a structured, organized, and complex group of sessile bacterial cells attached to a surface, which grow and interact as a community [1]. Bacterial biofilms are associated with human diseases, and account for 80% of bacterial chronic inflammatory and infectious diseases [2]. Compared to planktonic cells, biofilms are characterized by significant loss of susceptibility to antibiotics as well as high virulence potential [3], which explains why biofilms are associated with a tremendous impact on health, including increased morbidity and mortality [4]. Moreover, complications related to biofilms often result in additional hospitalization and medical care for patients, leading to substantial economic consequences [5].

*Staphylococcus aureus* and *Pseudomonas aeruginosa* are medically significant microbes that are capable of forming biofilms [3]. Methicillin-resistant *Staphylococcus aureus* (MRSA) is a major cause of hospital-acquired infections that are becoming more difficult to combat because of the development of bacterial resistance to most of the current antibiotics [6]. About 52.3% of intensive care unit nosocomial infections are due to MRSA [7]. As a result, biofilm formation contributes to the spread of MRSA in community and hospital settings. It also prolongs the duration of MRSA infection and colonization [8]. Similarly, *P. aeruginosa* is responsible for several opportunistic infections in immunocompromised patients. It is responsible for chronic lung infections in cystic fibrosis patients [9,10]. It is also the causative agent of urinary tract infections, septicemia, osteomyelitis, endocarditis [11], pneumonia, and burn infections [12].

The fluoroquinolones (FLQs) are a group of synthetic broad-spectrum antibiotics that inhibit bacterial cell replication by interfering with two enzymes that are essential in the process: DNA gyrase (topoisomerase II) and topoisomerase IV [13]. The FLQs are suitable for the treatment of urinary tract infections, gastrointestinal tract infections, respiratory tract infections, sexually transmitted infections, and skin infections [14,15]. They are effective on a variety of pathogens including *P. aeruginosa*, *Mycoplasma* spp., *Chlamydia* spp., *Staphylococcus* spp., and *Streptococcus* spp. [16]. The FLQs have several favorable properties, such as high potency, broad-spectrum activities, good tissue permeability, excellent bioavailability, and relatively few side effects. They are also available in both oral and intravenous formulations [17,18].

## 2. Materials and Methods

### 2.1. Bacterial Strains

Methicillin-resistant *Staphylococcus aureus* (MRSA) strain ATCC 43300 and *Pseudomonas aeruginosa* strain ATCC 27853 (Microbiologics, USA) were used in this study.

### 2.2. Antibacterial Agents

The following FLQs were used in this study: ciprofloxacin (Hikma Pharmaceutical: Amman, Jordan), gatifloxacin (Allergen Pharmaceuticals: Dublin, Ireland), levofloxacin (Sanofi-Aventis Deutschland GmbH: Frankfurt, Germany), moxifloxacin (Alcon Laboratories: Fort Worth, TX, USA), norfloxacin (Amman Pharmaceutical industries: Amman, Jordan), and ofloxacin (Allergen Pharmaceuticals, Ireland).

### 2.3. Antibacterial Susceptibility Test

The susceptibility of planktonic forms of MRSA and *P. aeruginosa* to the FLQs was assessed through the agar-well diffusion assay [19]. Sterilized Mueller-Hinton agar (MHA) plates were uniformly inoculated with the standardized bacterial suspension (1×10^8^ CFU/mL) using sterile cotton swabs, and then the plates were allowed to dry for five minutes. A sterile cork borer was used to make holes of 6 mm diameter in each plate. Wells were loaded with 100 μL of each antibiotic (stock solution concentration was 10 μg/mL, where all antibiotics were dissolved in distilled water), thus, equal concentrations of each drug were placed in each corresponding well. Negative control wells were loaded with 100 μL of sterile broth and bacterial suspension (no antibiotic). Each treatment was replicated three times and plates were incubated for 24 h at 37 °C. After the incubation period, the plates were examined for growth inhibition and the diameters of inhibition zones were measured using a standard ruler and recorded. In another experiment, the minimum inhibitory concentration (MIC) and minimum bactericidal concentration (MBC) values of the selected FLQs against both MRSA and *P. aeruginosa* planktonic forms were determined using the broth microdilution method [20]. Briefly, a two-fold serial dilution of the FLQs was carried out in a 96-well microtiter plate in accordance with the Clinical and Laboratory Standards Institute (CLSI) (2012) guidelines. A 100 μL aliquot of the adjusted bacterial suspension (5×10^5^ CFU/mL) was added into wells containing 100 μL of serially diluted antibiotics. Growth control and sterility control wells were inoculated with 200 μL of bacterial suspension and phosphate-buffered saline (PBS), respectively. The microtiter plate was then incubated at 37 °C for 18–24 h. Plates were assessed for growth visually and by measuring the optical density at 630 nm using a microtiter plate reader. Wells with the lowest antibiotic concentration that were visually clear and had an optical density of less than 0.1 were considered as the MIC for the selected FLQ. MBC was determined by inoculating 10 μL-aliquots from clear wells (of MIC of the particular antibiotic and higher) onto MHA. The plates were incubated at 37 °C for 18–24 h. The plates were examined for bacterial growth as described above for the MIC determination experiment, and the lowest antibiotic concentration that showed no growth was considered the MBC for the chosen antibiotic. For both MIC and MBC determination, a negative control was included, and it contained sterile broth and bacterial suspension (zero antibiotic concentration), and a positive control was chosen as the highest concentration of the particular antibiotics used.

### 2.4. Biofilm Assay and Determination of Minimum Biofilm Eradication Concentration (MBEC)

The assay was carried out using the Calgary 96-well Biofilm Device (Innovotech Inc.: Edmonton, AB, Canada) as previously described in [21]. Briefly, bacterial suspension was adjusted to 1×10^7^ CFU/mL. The biofilm device was inoculated by adding 150 μL of the inoculum into the wells of the 96 peg-lids on which the biofilm cells could build up. Sterility wells were inoculated with 150 μL of PBS. The pegs were incubated in a humidified incubator for 18–24 h under a rotation of 110 rpm at 37 °C to allow biofilm formation on the purpose-designed pegs. Once the biofilms were allowed to form, the pegs were rinsed with phosphate-buffered saline (PBS) to remove planktonic cells. Each peg-lid was then transferred into a ‘‘challenge 96-well microtiter plate’’ containing 200 μL of serially diluted antibiotics. Growth control wells and sterile control wells were filled with 200 μL of mycorrhiza helper bacteria (MHB) and PBS, respectively. The peg lids in the challenge plate were then incubated for 18–24 h at 37 °C. Finally, the peg lids were removed from the challenge plate and rinsed with PBS, then transferred into the recovery plate containing 200 μL of MHB in each well. The plate was incubated overnight at 37 °C.

To determine the MBEC values, the recovery plate was assessed visually for turbidity, and by measuring the optical density at 630 nm using a microtiter plate reader. Any visible growth indicated the detachment and re-growth of bacterial cells from the treated biofilms. The MBEC value represents the minimum antibiotic concentration that eradicates the biofilm following a suitable period of incubation [22]. Thus, the lowest antibiotic concentration that showed wells with no growth—visually clear with measured optical density of less than 0.1—was considered the MBEC for the selected antibiotic.

## 3. Results

### 3.1. Zones of Inhibition

The agar-well diffusion method was used to determine the zones of inhibition (ZOIs) of the selected FLQs against MRSA and *P. aeruginosa* planktonic cells. Among FLQs, moxifloxacin (33.8 mm) and gatifloxacin (32 mm) had the largest ZOIs against MRSA, while ciprofloxacin (30 mm) and gatifloxacin (22 mm) showed the highest ZOI diameters against *P. aeruginosa*. Table 1 shows each antibiotic with its respective ZOI measured in millimeters (mm). Data were obtained from three independent experiments.

### 3.2. Minimum Inhibitory Concentration (MIC)

The MIC of the selected FLQs was determined against both MRSA and *P. aeruginosa* planktonic strains using the broth microdilution method. Among FLQs, moxifloxacin (MIC = 0.049 µg/mL) and ciprofloxacin (MIC = 0.26 µg/mL) had the highest in vitro activity against MRSA and *P. aeruginosa,* respectively. The lowest MIC values were that of norfloxacin (1.172 µg/mL) against MRSA, and ofloxacin (3.33 µg/mL) against *P. aeruginosa* (Table 2).

### 3.3. Minimum Bactericidal Concentration (MBC)

The MBCs of the six FLQs were determined to assess the concentrations of these FLQs that can permanently kill the planktonic cells. All six FLQs had MBC values that were always higher than their MIC, indicating that these antibiotics are not bactericidal at the MIC. The only exception was gatifloxacin, which had an MBC value that was identical to its MIC against *P. aeruginosa* (Table 2).

### 3.4. Minimum Biofilm Eradication Concentration (MBEC)

The MBEC values of the selected FLQs were determined using the Calgary Biofilm Device. The term MBEC refers to the concentration that prevents the regrowth of the bacteria following an overnight incubation in a recovery media of the antibiotic treated biofilms [22]. Gatifloxacin (328.13 µg/mL) and moxifloxacin (390.63 µg/mL) were the most potent against MRSA biofilm. On the other hand, ciprofloxacin (40 µg/mL) was the most potent against *P. aeruginosa* biofilm, followed by norfloxacin, which had an MBEC value of 160 µg/mL. Norfloxacin (875 µg/mL) and ofloxacin (640 µg/mL) were the least potent against MRSA and *P. aeruginosa* biofilms, respectively (Table 3).

### 3.5. Comparison of Antibacterial Activities of Fluoroquinolones

In this study, we demonstrated that the activities of the six FLQs varied between planktonic and biofilm cells. Moxifloxacin and gatifloxacin were the most potent against both planktonic and biofilm MRSA bacteria, however, not in the same order of activity. Norfloxacin was the least active when tested against both planktonic and biofilm cells. Against *P. aeruginosa*, ciprofloxacin was the most potent on both planktonic and biofilm cells, and ofloxacin remained the least potent on both biofilm and planktonic cells. However, gatifloxacin, levofloxacin, moxifloxacin, and norfloxacin showed moderate activity that differed between the planktonic and biofilm cells. Table 4 shows the in vitro order of decreasing activity of the selected FLQs.

## 4. Discussion

In this confirmatory study, the susceptibility pattern of *P. aeruginosa* towards the selected FLQs was assessed through the determination of zones of inhibition using the agar-well diffusion method. The data obtained from the current study revealed that all the selected FLQs showed considerable growth inhibitory zones, although the susceptibility of *P. aeruginosa* to these antibiotics varied from high to moderate. Ciprofloxacin measured the lowest MIC among all other FLQs, which is in agreement with previous studies [11,23]. For other FLQs (i.e., gatifloxacin, norfloxacin, and levofloxacin), MICs showed that they were equally potent against *P. aeruginosa*, which is again in confirmation of previous studies [24]. Furthermore, *P. aeruginosa* was found to have improved susceptibility to levofloxacin as compared to *Escherichia coli*, *Staphylococcus aureus*, and *Klebsiella pneumoniae* [25]. Based on data obtained from zones of growth inhibition of *P. aeruginosa*, FLQs can be listed in order of decreasing anti-pseudomonal activity as ciprofloxacin, gatifloxacin, norfloxacin, levofloxacin, moxifloxacin, and ofloxacin. A similar conclusion was attained in a previous study of the susceptibility of *P. aeruginosa* to five FLQs using the disc diffusion method, where it was reported that ciprofloxacin was the most potent among FLQs followed by levofloxacin, norfloxacin, and lastly ofloxacin [26].

Regarding the MBC, all six FLQs had MBC values that were approximately two-fold higher than their MICs. This indicates that the bactericidal (killing effect) of these agents is achieved at a higher concentration than the MIC. The only exception was gatifloxacin, which had similar MIC and MBC values against *P. aeruginosa*. An early published study reported that ciprofloxacin had Minimum Bactericidal Concentration 50 (MBC50) and Minimum Bactericidal Concentration 90 (MBC90) values that were twice as large as its Minimum Inhibitory Concentration 50 (MIC50) and Minimum Inhibitory Concentration 90 (MIC90), respectively, [27] against MRSA, which is consistent with current findings. A similar conclusion was observed in a later study stating that the MBC50s of ciprofloxacin, ofloxacin, and norfloxacin were two times greater than their MIC50 values [28]. In spite of the previous facts, FLQs are still regarded as bactericidal according to the NCCLS (1999) criteria, which defines the bactericidal activity as a ratio of MBC to MIC of greater than four.

The susceptibility study of *P. aeruginosa* biofilm to the selected FLQs was carried out by the use of the Calgary Biofilm Device to determine the minimum biofilm eradication concentration (MBEC). Several studies have indicated that biofilm bacteria are less susceptible than their planktonic counterparts [3,29,30]. Ciprofloxacin was shown to be the most active against biofilm cells, having an MBEC of 40 µg/mL, which is more than 100-fold higher than its MIC for planktonic cells. A slightly higher MBEC of 64 µg/mL was previously reported [3]. Such deviations can be accounted for by variations in the density of biofilms exposed to the antibiotic, which depends on the adopted method for biofilm formation. In the case of norfloxacin (MBEC: 160 µg/mL), the concentration was almost 200× the MIC, and levofloxacin concentration (MBEC: 320 µg/mL) was also more than 300× the planktonic MIC. Likewise, the concentration needed for gatifloxacin (MBEC: 533.33 µg/mL) was more than 800× its MIC. These findings are in accordance with an earlier publication that stated that bacterial biofilms can be up to 1000-fold less susceptible than planktonic cells [31]. As evident from this study, ofloxacin (MBEC: 640 µg/mL) is the least potent fluoroquinolone against *P. aeruginosa*. In that respect, current results contradict other works that reported ofloxacin as a potent antibiotic against *P. aeruginosa* biofilms [32,33].

For MRSA, the newer third generation FLQs (gatifloxacin and moxifloxacin) again showed the most promising results by having the lowest MBEC values among the selected FLQs. The MBECs for gatifloxacin and moxifloxacin were 328.13 μg/mL and 390.63 μg/mL, respectively. In agreement with this finding, a recent study concluded that moxifloxacin treatment in an in vitro model exhibited superior anti-biofilm activity against MRSA and methicillin-resistant *Staphylococcus epidermidis* biofilms compared to vancomycin [34]. Norfloxacin had the highest MBEC (875 μg/mL), and thus was the least effective in vitro against both planktonic and biofilm bacterial cells.

It is evident from the results obtained that norfloxacin and ofloxacin were the least potent against both planktonic and biofilm cells of MRSA, while ciprofloxacin and ofloxacin were the most potent and the least potent, respectively, against both planktonic and biofilm cells *of P. aeruginosa*. However, the efficacy of the remaining selected FLQs on planktonic cells did not correspond to that on biofilm cells. For instance, levofloxacin was more effective against planktonic cells of MRSA than on biofilms, while ciprofloxacin was more effective against biofilms than planktonic cells. A similar trend applied to moxifloxacin and gatifloxacin against MRSA, and gatifloxacin and norfloxacin against *P. aeruginosa*. This was compatible with the fact that certain antibiotics are more effective against biofilms of *P. aeruginosa* than other antibiotics [29,30,35,36,37,38]. For example, ciprofloxacin and ofloxacin were more effective against biofilms of *P. aeruginosa* than other antibiotics such as ceftazedim, gentamicin, imipenem, amikacin, azithromycin, and erythromycin [32]. This difference in antibiotic susceptibility could be related to differences in bacterial behavior in the biofilm state as compared to that in the planktonic form. It could also be related to the chemical structures of respective antibiotics, where ciprofloxacin and norfloxacin differ in only a single chemical moiety, and moxifloxacin and gatifloxacin similarly differ in only one chemical moiety. Further analysis of the effect of the relationship of chemical structure to the activities of FLQs against bacteria in the biofilm state could be the matter of a separate future study which would take into account previous work ranging from X-ray crystallography to resistance to toxic side effects [39,40,41]. In that respect, it could be the case that some FLQs are more rapidly lethal, and if so, it is apparent that only the most effective FLQs should be used, since the weaker ones will select for resistance and ruin the entire class. For example, the quinolones induce the SOS response, which is mutagenic. Thus, FLQs that are highly lethal are expected to restrict the emergence of resistance better than ones that are less lethal or less potent [42,43].

The order of decreasing antibacterial activity corresponding to the diameter zones of inhibition was consistent with that found from our MIC measurements. Another issue to be considered is the basis for biofilm eradication, and the relationship between MIC and the lethal activity, especially rapid killing (not MBC). They appear to involve different mechanisms. Where the MIC is due to trapping gyrase on DNA, rapid killing arises from the release of DNA breaks and the accumulation of reactive oxygen species [44]. The MBC, on the other hand, could be reflecting other secondary reactions. More work is needed toward this end [44].

In the future, in vivo clinical studies are required for the proper assessment of the safety and efficacy of these FLQs against biofilms. An earlier study [45] reported that moxifloxacin retained a low MIC against ciprofloxacin-resistant strains of *Staphylococcus aureus*, although treatment with this antibiotic failed when tested against experimental models of endocarditis. This could be related to the fact that while the primary target for ciprofloxacin is topoisomerase IV, it is DNA gyrase for moxifloxacin [46,47]. Thus, it seems plausible that moxifloxacin will retain some activity in ciprofloxacin-resistant strains. Yet, and for unknown reasons, the resistance to one of these targets increases the probability that resistance will develop in the other target [48,49]. Other studies reported that FLQs may increase the risk of MRSA in hospitalized patients [50,51]. On the other hand, previous studies also reported that FLQs have potent antibacterial activities against *P. aeruginosa* biofilms [32,52]. Furthermore, the best antibiotic against *P. aeruginosa* among this class of antimicrobials was ciprofloxacin [18].

Taken together, current results are confirmatory of previous works and indicate that *P. aeruginosa* and MRSA are susceptible (either highly or moderately) to all the FLQs that were tested here, and ciprofloxacin remains the most effective.

## Figures and Tables

**Table 1:** Zone of inhibition of the selected fluoroquinolones (FLQs) against methicillin-resistant *Staphylococcus aureus* (MRSA) and *Pseudomonas aeruginosa* planktonic cells.

Fluoroquinolone	Diameter of the Zone of Inhibition (mm) against MRSA	Diameter of the Zone of Inhibition (mm) against *P. aeruginosa*
Ciprofloxacin	22.8 ± 0.8	30.3 ± 0.6
Gatifloxacin	32.0 ± 0.0	22.3 ± 0.6
Levofloxacin	26.3 ± 0.6	21.0 ± 1.0
Moxifloxacin	33.8 ± 0.3	19.3 ± 1.5
Norfloxacin	10.6 ± 0.5	21.3 ± 1.2
Ofloxacin	22.3 ± 0.3	16.0 ± 1.0

Data are represented as mean ± standard deviation.

**Table 2:** Minimum inhibitory concentration (MIC) and minimum bactericidal concentration (MBC) values of FLQs against MRSA and *P. aeruginosa* planktonic cells.

	MRSA		*P. aeruginosa*	
Fluoroquinolone	MIC (µg/mL)	MBC (µg/mL)	MIC (µg/mL)	MBC (µg/mL)
Ciprofloxacin	0.235 ± 0.084	0.625 ± 0.00	0.26 ± 0.09	0.31 ± 0.00
Gatifloxacin	0.078 ± 0.00	0.156 ± 0.00	0.63 ± 0.00	0.63 ± 0.00
Levofloxacin	0.156 ± 0.00	0.313 ± 0.00	1.04 ± 0.36	1.25 ± 0.00
Moxifloxacin	0.049 ± 0.018	0.0781 ± 0.00	1.67 ± 0.72	2.5 ± 0.00
Norfloxacin	1.172 ± 0.221	2.5 ± 0.00	0.83 ± 0.36	1.25 ± 0.00
Ofloxacin	0.352 ± 0.11	0.625 ± 0.00	3.33 ± 1.44	5.00 ± 0.00

Data are represented as mean ± standard deviation.

**Table 3:** Minimum biofilm eradication concentration (MBEC) values of FLQs against biofilm cells of MRSA and *P. aeruginosa.*

Fluoroquinolone	MBEC (µg/mL) against MRSA Biofilm	MBEC (µg/mL) against *P. aeruginosa* Biofilm
Ciprofloxacin	438 ± 125	40 ± 0
Gatifloxacin	328 ± 94	533 ± 185
Levofloxacin	625 ± 0	320 ± 0
Moxifloxacin	390 ± 156	427 ± 185
Norfloxacin	875 ± 250	160 ± 0
Ofloxacin	750 ± 0	640 ± 0

Data are represented as mean ± standard deviation.

**Table 4:** Comparison of antibacterial activities of FLQs in decreasing order.

	Against Planktonic Cells		Against Biofilm	
Order of Activity	MRSA	*P. aeruginosa*	MRSA	*P. aeruginosa*
1	Moxifloxacin	Ciprofloxacin	Gatifloxacin	Ciprofloxacin
2	Gatifloxacin	Gatifloxacin	Moxifloxacin	Norfloxacin
3	Levofloxacin	Norfloxacin	Ciprofloxacin	Levofloxacin
4	Ciprofloxacin	Levofloxacin	Levofloxacin	Moxifloxacin
5	Ofloxacin	Moxifloxacin	Ofloxacin	Gatifloxacin
6	Norfloxacin	Ofloxacin	Norfloxacin	Ofloxacin
